# IL40: A Newly Described Cytokine With Conflicting Measurements and Detection Variability—Are There Different Forms?

**DOI:** 10.1111/sji.70105

**Published:** 2026-03-08

**Authors:** Nora Euler, Wenqi Huang, Peter Hemmingsson, Anna Juto, Erik Hellbacher, Lars Klareskog, Vivianne Malmström, Iva Gunnarsson, Eva Baecklund, Caroline Grönwall

**Affiliations:** ^1^ Division of Rheumatology, Department of Medicine/Solna, Center for Molecular Medicine Karolinska Institutet and Karolinska University Hospital Stockholm Sweden; ^2^ Department of Medical Sciences, Rheumatology Uppsala University Uppsala Sweden

**Keywords:** ANCA‐associated vasculitis, biomarker measurements, *C17orf99*, DLBCL, IL40, rheumatoid arthritis

## Abstract

Interleukin‐40 (IL40) is a recently described 27 kDa cytokine encoded by *C17orf99*, originally suggested to play a role in B‐cell biology, but its function is largely unknown. However, elevated serum levels have been reported in rheumatic diseases. Most published studies focus on IL40 measurements in serum/plasma using commercial sandwich ELISAs. Here we found large discrepancies between two IL40 ELISAs (Mybiosource and Abbexa), with Abbexa reporting significantly higher plasma levels. In our investigation, IL40 (Abbexa) was not elevated in patients with ANCA‐associated vasculitis, early or established rheumatoid arthritis (RA), or RA patients who had developed B cell lymphoma (RA‐L), compared to healthy donors. Yet, we found significant correlation of Abbexa IL40 levels with BAFF and APRIL. We next compared the binding of IL40 between the two commercial assays. Pre‐adsorption experiments showed that the Mybiosource capture antibody bound the same target as the Abbexa capture antibody but did not detect the same IL40. Moreover, neither assay detected the reciprocal IL40 kit reference nor mammalian expressed recombinant IL40. In contrast, a Human Protein Atlas (HPA) antibody towards the unstructured C‐terminal of IL40, despite being only partly validated by HPA, detected recombinant IL40 in Western blot and ELISA. We speculate that there may be different structural or modified forms of IL40. The discrepancy between the IL40 Abbexa results and the literature also highlights the difficulties in interpreting results from commercial antibodies and assays.

## Graphical abstract

IL40 has previously been reported to be elevated in rheumatic disease. Here we did not observe higher IL40 plasma levels in rheumatoid arthritis (RA) patients or ANCA‐associated vasculitis (AAV) patients compared to healthy donors and further found large discrepancies in IL40 measurements by two commercial ELISAs.

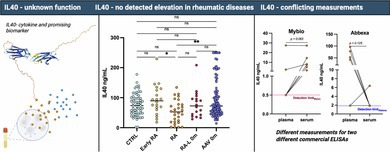

## 1 Introduction

Interleukin‐40 (IL40) is encoded by the gene C17ORF99 (chromosome 17 open reading frame 99) found exclusively in mammalian genomes and resulting in a 27 kDa protein (29 kDa when including the secretory signal). IL40 was first described as a novel cytokine in 2017 by Catalan‐Dibene et al. [1] While IL40 has gained increasing interest recently as an inflammatory biomarker, its function remains relatively elusive. In fact, IL40 does not share high homology with any other protein, and so far no receptor binding for IL40 has been identified. IL40 has been reported to be expressed by stromal cells [1], activated B cells [1] and neutrophils [2], and with high expression in the bone marrow and lymphoid tissue according to the Human Protein Atlas mapping [3, 4]. Knockout mice suggest that this protein is important in B cell homeostasis, as IL40^−/−^ mice have been shown to have a reduced number of progenitor and mature B cells in the bone marrow and to have significantly lower frequency of class‐switched IgA B cells in gut‐associated lymphoid tissue, as well as lower levels of secreted IgA in serum, faeces and milk [1]. A recent study also found that IL40^−/−^ knockout mice had reduced fatality during sepsis, suggesting a relevance in inflammatory responses [5].

In rheumatic diseases, IL40 has been reported to be elevated in serum from patients with rheumatoid arthritis (RA), Sjögren disease and systemic sclerosis, compared to healthy donors [6, 7, 8, 9]. Furthermore, high concentrations of IL40 have been detected in synovial fluid from RA patients and osteoarthritis patients [10]. In RA, serum and synovial fluid IL40 concentrations have been shown to correlate with hallmark diagnostic autoantibodies such as anti‐CCP2 IgG (anti‐cyclic citrullinated peptide antibodies) and rheumatoid factor IgM (RF IgM) levels. Furthermore, IL40 measurements in synovial fluid from RA patients have been shown to correlate with inflammatory markers associated with neutrophil activity (e.g., IL‐8, MIP‐1α, PR3) and measurements associated with disease activity [7]. Indeed, in vitro studies support that IL40 can promote the expression of inflammatory markers and the formation of neutrophil extracellular traps (NETs) in connective tissue cells [7, 10]. IL40 has also been shown to be produced by certain lymphoma cell lines, and to be expressed in tumour tissue from patients with different malignancies including lymphoma [11, 12], suggesting that this protein may also be relevant to study in the context of cancer.

The majority of studies measuring IL40 in serum/plasma have used the same commercial ELISA from Mybiosource (cat.no MBS9337680). In this study, we compared IL40 measurements with another commercial assay from Abbexa (cat.no abx508530) and observed significant differences in the measured plasma and serum levels and their association with inflammatory conditions in patients with rheumatoid arthritis (RA). This opens up for either cross‐reactivity to other protein or the detection of a distinct conformational form of IL40. If there are different forms of IL40, these may have different biological roles and thereby clinical implications, making it important to highlight and report these differences for future studies of IL40.

## 2 Material and Methods

### 2.1 Study Populations

In this study we included rheumatoid arthritis patients with early disease (Early RA, *n* = 20) from the EIRA study (Epidemiological Investigation of Rheumatoid Arthritis) and healthy population controls from the EIRA study (CTRL, *n* = 61) [13, 14]. We also included patients with established RA (RA, *n* = 29) recruited from the Karolinska University Hospital (Stockholm, Sweden) and patients with longstanding RA and a subsequent diagnosis of diffuse large B‐cell lymphoma (RA‐L, *n* = 18) included from the Swedish AUTOLYMPHOMA cohort study previously described [15]. All RA patients fulfilled the 2010 ACR/EULAR RA classification criteria [16]. In addition, we also included ANCA‐associated vasculitis patients (AAV, *n* = 98) recruited at the Karolinska Hospital before treatment and at 6‐month follow‐up after initiating treatment. Among the AAV patients, 60% received rituximab and 40% received cyclophosphamide as treatment. All AAV patients included had kidney involvement and were assessed to have low disease activity at 6‐month follow up after treatment. The RA‐L cohort plasma samples were analysed from the time of lymphoma diagnosis, before rituximab chemotherapy combination treatment (cyclophosphamide, doxorubicin, vincristine and prednisone; R‐CHOP‐like regimens) (0 m, baseline), as well as from 6‐, 12‐ and 24 months after lymphoma diagnosis and initiation of lymphoma treatment. All serum and plasma samples were stored at −80°C.

### 2.2 IL40 Measurements

IL40 was measured in EDTA‐plasma at baseline for all patient groups and the population controls included in this study, and at follow‐up after treatment for AAV (6 m) and RA‐L patients (6, 12 and 24 m) using ELISA (Abbexa, abx508530) according to the manufacturer's instructions at 1:5 dilution. 1:20 dilution was also evaluated for some samples. IL40 in both serum and plasma was also measured in a selected number of samples using the Mybiosource IL40 assay (MBS9337680) following the manufacturer's instructions. Samples for in‐depth comparisons were selected based on Abbexa IL40 plasma results and included six RA patients, whereof two with IL40 measurements below detection (1.9 ng/mL), four with values close to the median measurement in RA and four with values above the median value. Two population controls with low IL40 concentrations were also included.

### 2.3 IL40 ELISA Pre‐Adsorption

Plasma samples were diluted [1:5] and added to the pre‐coated ELISA from either Abbexa or Mybiosource for 1 h pre‐incubation at room temperature, and thereafter the plasma was transferred to the other assay (i.e., preincubated plasma from Abbexa was added to pre‐coated wells in the Mybiosource assay, vice versa). In total, 8 of the plasma samples (6 RA patient samples, 2 population controls) were used for the pre‐adsorption to determine whether the two assays detect the same IL40. Plasma (1:5 and 1:20 dilution) and serum (1:5 dilution) from the same patients included in the pre‐incubation was also measured using the standard protocol from both assays, respectively. Both IL40 kits provide a reference for quantification that is based on *E. coli* expressed recombinant protein, for the Abbexa assay equivalent to human IL40 (Q6UX52) aa21‐265. The range of detection for the Abbexa kit was 0.78–50 ng/mL and 0.25–8 ng/mL in the Mybiosource assay. We also evaluated binding to HEK293 produced recombinant IL40, expressed as a fusion protein with C‐terminal Fc (Creative Biomart, C17ORF99‐93H) at two different concentrations (5 ng/mL and 2.5 ng/mL) in each assay. We also tested the binding of the IL40 reference, also known as standard, from Mybiosource on the Abbexa detection assay and vice versa to compare binding. The IL40 standards provided by the two different assays are both recombinant full‐length protein (UniProt: Q6UX52) produced in *E. coli* according to the information given by the manufacturers.

### 2.4 Additional Measurements of Inflammatory Markers and Autoantibodies

IL40 plasma measurements in patients with established RA, RA with lymphoma and population controls were compared to cytokine and chemokine plasma measurements, RA associated autoantibodies (CCP2 IgG, RF) and C‐reactive protein (CRP) levels in serum. In total 12 different cytokines and chemokines were measured in plasma using a custom Luminex assay: TNF, IL‐6, IL‐10, IL‐8, CCL3, CCL4, CXCL9, CXCL10, APRIL, BAFF, IL‐21 and CXCL13 as previously described [15]. C‐reactive protein (CRP) levels were measured by a high‐sensitivity ELISA (Thermo Fisher Scientific, human CRP instant ELISA). CCP2 IgG was measured using IMMUNOSCAN CCPlus ELISA (Svar Life Science) and rheumatoid factors, abbreviated RF (RF IgM, RF IgG, RF IgA), were measured using QUANTA Lite ELISAs (Werfen). The unrelated inflammation‐associated anti‐modified protein autoantibodies anti‐MDA‐modified protein IgG, IgM, IgA were measured in serum using an in‐house ELISA protocol as previously described [17, 18, 19].

### 2.5 Western Blot and Dot Blot Analysis

To further evaluate the Abbexa anti‐IL40 detection reagent, Western blot analysis was performed. 4 μL plasma diluted 1:20 from a patient with high IL40 levels (112 ng/mL) according to the Abbexa assay as well as plasma from another individual with low levels (1.9 ng/mL, below detection limit) that were run separately under reducing and non‐reducing conditions on SDS‐PAGE, Bolt 2%–12% bis‐tris gel with MES SDS‐running buffer and transferred to PVDF (0.2 μm) membranes (Thermo Fisher Scientific) according to the manufacturer's instructions. We also dissolved the Abbexa standard in PBS and separated a volume that would be equivalent to 10 ng IL40/lane. Recombinant Fc‐IL40 (40 or 10 ng/lane, Creative Biomart) was analysed in parallel. A separate SDS‐PAGE as described above was performed for recombinant Fc‐IL40 (400 ng/lane) with reduced and non‐reduced conditions and stained with SimplyBlue SafeStain Coomassie (Thermo Fisher Scientific). We also performed a dot blot, where recombinant IL40, Abbexa reference sample, or plasma were directly applied to a nitrocellulose membrane. Membranes were blocked with 3% BSA in PBS for 1 h, followed by incubation with the Abbexa reagent A (biotinylated rabbit anti‐IL40) at 4°C overnight and detection with Abbexa reagent B (HRP‐Streptavidin) diluted according to the manufacturer's instructions for the ELISA kit. Signals were detected with chemiluminescence Clarity Western ECL substrate (Bio‐Rad). Detection was compared to an anti‐IL40 antibody generated within Human Protein Atlas efforts (HPA, rabbit anti‐IL40 HPA029655) [20] at 1:500 dilution in 1% casein in PBS, followed by HRP conjugated goat anti‐rabbit IgG (Cell Signaling Technology). Importantly, the HPA antibody has only been partially validated by Human Protein Atlas and did not pass all quality control steps. While showing high antibody specificity by protein array, it has not passed quality control testing for Western blot and the results from immunohistochemistry were not consistent with RNA transcript data.

### 2.6 ELISA Comparing HPA and Abbexa Anti‐IL40

To further investigate the Abbexa IL40 recognition we compared the Abbexa antibody with the HPA anti‐IL40 (HPA029655). Mammalian produced recombinant IL40‐Fc (Creative Biomart) was coated in PBS in high‐binding half‐area plates (Corning) at different concentrations with the highest at 1000 ng/mL. Plates were blocked with 3% BSA in PBS before addition of HPA anti‐IL40 or Abbexa detection antibody (reagent A) diluted in Abbexa diluent A at 1:1000 or 1:100, respectively and incubation for 1.5 h at 37°C. Binding was detected using HRP goat anti‐rabbit HPR (Cell signaling Technology) and TMB substrate (Biolegend). We also used HPA anti‐IL40 coated wells for pre‐adsorption before Abbexa detection, similarly as described above. In addition, an experiment was performed where the Abbexa pre‐coated kit plate was exchanged for an HPA anti‐IL40 coated and blocked plate, but all subsequent steps, sample dilution and detection reagents were in accordance to the Abbexa kit.

### 2.7 Statistical Analysis

The differences in concentration of IL40 in RA, early RA, RA‐L and AAV were compared using the Kruskal Wallis test adjusted for multiple comparison using Dunn's method. IL40 measurements before and after treatment for RA‐L patients (0 m vs. 6 m, 0 m vs. 12 m, 0 m vs. 24 m) and AAV (0 m vs. 6 m) were compared using the Wilcoxon matched pairs signed rank test. Comparison of IL40 plasma concentrations determined by the Abbexa assay and Mybiosource assay was done by the Wilcoxon matched pairs signed rank test for RA patient samples (*n* = 6). To investigate whether there is a correlation between IL40 plasma measurements by the two different commercial assays, Spearman correlation was applied. Significance was set to a *p*‐value < 0.05. Statistical analysis was performed using Prism 10 (GraphPad) or R version 4.5.2.

## 3 Results

### 3.1 Clinical Significance of IL40 Abbexa Measurements

**FIGURE 1:**
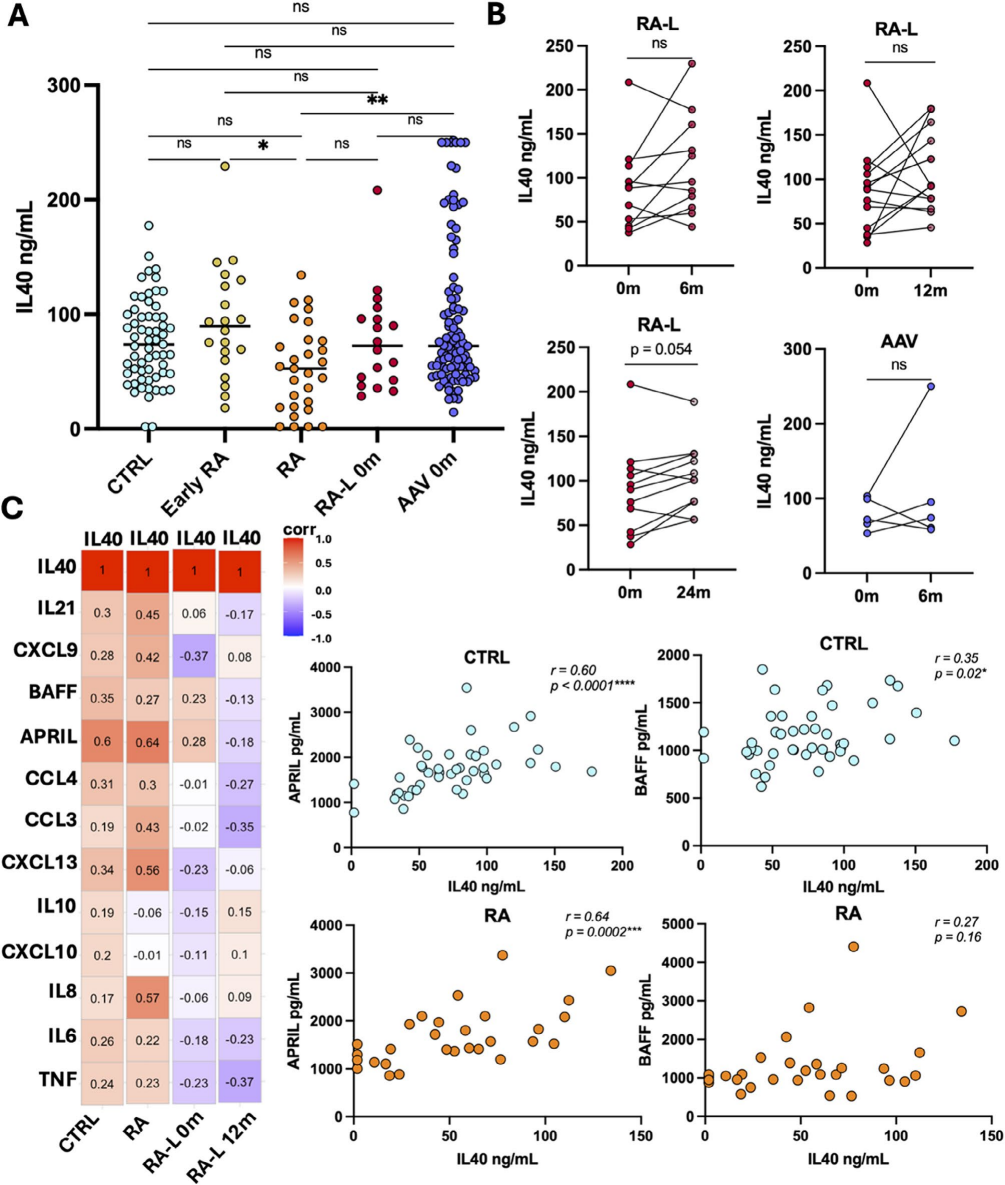
Plasma IL40 levels measured by Abbexa assay are high in healthy donors and correlate with APRIL & BAFF. (A) Abbexa IL40 plasma concentrations (ng/mL) in healthy population controls (CTRL, *n* = 61), patients with recently diagnosed rheumatoid arthritis (Early RA, *n* = 20), rheumatoid arthritis patients with established disease (RA, *n* = 29), rheumatoid arthritis patients with diffuse large B cell lymphoma as a comorbidity (RA‐L, *n* = 18) and ANCA‐associated vasculitis patients (*n* = 98). Statistical comparison was done by Kruskal Wallis test adjusted for multiple comparisons using Dunn's method. (B) Abbexa IL40 plasma concentrations (ng/mL) at baseline and at follow‐up after treatment for RA‐L patients (6‐, 12‐, 24‐month after lymphoma diagnosis and initiation of treatment) and AAV patients (6‐month following initiation of treatment). Wilcoxon matched paired test was applied to compare IL40 measurements before and after treatment for both patient groups. (C) Spearman correlation matrix for Abbexa IL40 plasma concentrations with 12 other cytokines and chemokines measured previously by Luminex. Correlation analysis was performed for the healthy donors (*n* = 45), RA patients (*n* = 29) and RA‐L patients at baseline (0 m, *n* = 18) and after treatment (12 m, *n* = 11). For all statistical analysis, a *p*‐value < 0.05 was considered significant. Significance written as *p < 0.05, **p < 0.01, ***p < 0.001, ****p < 0.0001.

Our data demonstrate high IL40 plasma concentrations in both patients with rheumatic disease and healthy population controls when analysed with the Abbexa assay. Interestingly, untreated early RA and ANCA‐associated vasculitis (AAV) patients displayed higher levels of IL40 compared to RA patients with established disease (*p* = 0.035 and *p* = 0.0072, respectively) (Figure 1A). We also had the opportunity to analyse IL40 in a unique subset of RA patients that had developed diffuse large B‐cell lymphoma (DLBCL; RA‐L). No significant difference in IL40 concentrations was observed for RA‐L patients compared to other RA patient groups or compared to patients with AAV or healthy donors. No significant difference in IL40 levels was found before and after lymphoma rituximab combination treatment for RA‐L patients at 6‐, 12‐ and 24‐month follow‐up. Similar results were observed for AAV patients when comparing IL40 concentrations at baseline to 6‐month follow‐up samples (Figure 1B). However, we observed a significant correlation of IL40 with APRIL for both RA patients and healthy donors, and correlations for IL40 with BAFF for the healthy donors (Figure 1C). No correlation was found for APRIL and BAFF for the RA‐L patients at baseline or at follow‐up after rituximab treatment. No significant correlation was observed for IL40 with any of the other measured cytokines and chemokines for the patient groups included or healthy controls. Further investigation of the association between IL40 and hallmark RA autoantibodies and CRP levels did not reveal any significant correlations (Figure S1). Our data was in striking contrast to previous findings from other groups based on the Mybiosource methodology, which reported significantly higher IL40 in rheumatic disease compared to healthy controls. The total levels that we detected were also quantified as significantly higher compared to previous reports [2, 7, 9, 21]. Notably, the included healthy population controls were sex and age matched with the RA patients and had no rheumatic disease. CRP assessment confirmed that they had no indication of systemic inflammation (Figure S2). We also did not see any association for the IL40 levels with repeated freeze‐thawing or any so‐called hook effect if the samples were assessed at a higher dilution factor (Figure S3).

### 3.2 Different Measurements of Plasma IL40 Using the Mybiosource Assay

**FIGURE 2:**
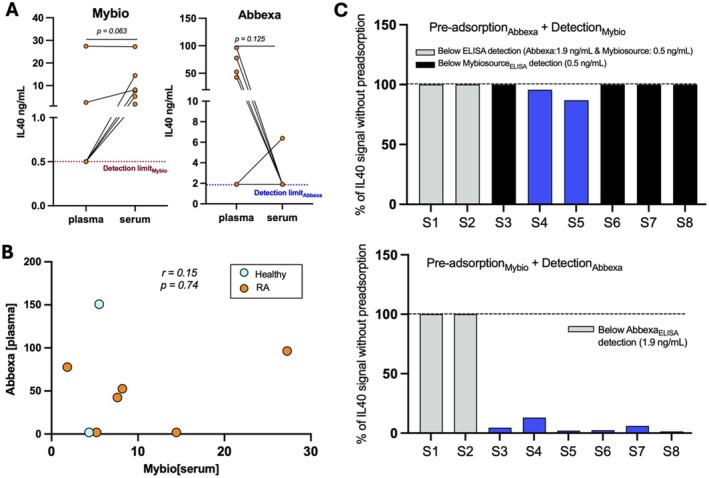
Comparing Abbexa and Mybiosource commercial IL40 kits reveals significant differences. (A) Differences in IL40 concentrations (ng/mL) in plasma versus serum by the Mybiosource assay (Mybio, left) and Abbexa assay (right) were compared in six RA patients using the Wilcoxon matched paired test. (B) Spearman correlation analysis for IL40 plasma concentrations determined by Abbexa versus IL40 measurements in paired samples using the Mybiosource assay (right). In total, six RA samples and two healthy donor samples were included. (C) The results of the pre‐adsorption ELISA experiment are shown as the percentage of IL40 measurement without preadsorption for each assay. In total, eight samples (S1–S8) were included, six from RA patients and two from healthy donors (see methods). For all statistical analysis, a *p*‐value < 0.05 was considered significant.

The relatively high IL40 plasma levels measured by the Abbexa assay were surprising, therefore we also compared parallel measurements in a selection of samples using the assay by Mybiosource (MBS9337680). Notably, the IL40 concentrations determined by the two different assays correlated poorly with each other and the measurements were significantly lower when using the Mybiosource assay compared to the Abbexa assay (Figure 2A,B). Per recommendation by the manufacturers, we primarily measured IL40 in plasma samples. However, we also had access to paired serum and plasma samples from six RA patients with established disease sampled at the same timepoint (preselected based on Abbexa plasma measurements, see methods), and direct comparison showed lower IL40 in serum vs. plasma by the Abbexa assay, but higher IL40 in serum vs. plasma by the Mybiosource assay. Notably, the majority of IL40 measurements fell below the kit detection threshold when using plasma in the Mybiosource assay (< 0.5 ng/mL), and serum in the Abbexa assay (< 1.9 ng/mL) (Figure 2A). The corresponding optical density (OD) output value for IL40 measurements showed an OD‐value close to zero for Mybiosource plasma measurements and Abbexa serum measurements, respectively. Since the majority of IL40 plasma measurements fell below the detection range of the Mybiosource assay (0.5 ng/mL), correlation analysis for the IL40 measurements were conducted for serum samples. Correlation analysis of IL40 serum levels measured by Mybiosource for the six preselected RA patients showed a trend for a correlation with hallmark RA autoantibodies RF IgM (*r* = 0.83, *p* = 0.06), RF IgG (*r* = 0.83, *p* = 0.06), RF IgA (*r* = 0.71, *p* = 0.14) and anti‐CCP2 IgG (*r* = 0.77, *p* = 0.11) (Figure S1). No correlation was observed for IL40 serum measurements by Mybiosource and C‐reactive protein levels (*r* = −0.26, *p* = 0.66). While not significant, IL40 serum concentrations by Mybiosource showed relatively high correlation with anti‐MDA IgM (*r* = 0.60, *p* = 0.24), anti‐MDA IgG (*r* = 0.71, *p* = 0.14) and anti‐MDA IgA (*r* = 0.77, *p* = 0.11) (Figure S1). When comparing correlations for IL40 with inflammatory associated cytokines and chemokines, we observed a significantly negative correlation for serum IL40 with BAFF in RA patients (*r* = 1.00, *p* = 0.02) (Figure S4). No other significant correlations were observed for IL40 with other cytokines/chemokines; however, some markers had a trend for a negative correlation. Taken together, this implicated differences in the detection between the two assays.

### 3.3 Comparison of Two Different Commercial Assays for Detection of Soluble IL40

Since there were large differences observed in IL40 measurements between the Abbexa assay and Mybiosource assay, we next performed a pre‐adsorption experiment that compared the capture and detection of IL40 in the two commercial assays (Abbexa: in our studies, Mybiosource: previous studies). When plasma samples were pre‐incubated on Mybiosource coated wells before transfer to the Abbexa kit and detection with the Abbexa reagents, we could observe an 87%–99% reduction in the IL40 levels for six of the eight pre‐incubated samples (Figure 2C). Two samples (S1 and S2) did not change but these displayed IL40 levels below the limit of detection (1.9 ng/mL). Hence, the Mybiosource capture antibody binds to the same IL40 protein as detected by the Abbexa assay. However, in contrast, the detection in the Mybiosource assay did not markedly change by pre‐adsorption to the Abbexa coated wells (Figure 2C). However, it should be noted that six of the eight samples included were below the detection limit of the assay. Importantly, the two different commercial assays could not detect the respective IL40 standard (Figure S5), despite both representing the full‐length recombinant IL40 protein and both being produced in *E. coli* (Table 1). Furthermore, neither ELISA kit detected the recombinant HEK293 produced Fc‐IL40 protein (Figure 2D). Yet, it should be acknowledged that the Fc‐IL40 will occur in a forced dimeric form due to the Fc‐conjugation.

**TABLE 1:** Overview of investigated anti‐IL40 capture and detection antibodies.

Antibody	Abbexa (abx508530)		Mybiosource (MBS9337680)		Human protein atlas (HPA029655)
	Capture	Detection	Capture	Detection	Detection
Immunogen^a^	aa 21–265	aa 21–265	Full‐length protein	aa 21–214	aa 190–265
Host	Rabbit	Rabbit	Mouse	Rabbit	Rabbit
Antibody type	Polyclonal	Polyclonal	Monoclonal	Polyclonal	Polyclonal

^a^ Residue number based on Unipot Acc No. Q6UX52.

### 3.4 Western Blot and ELISA Using HPA Anti‐IL40 Reveals Differences in IL40 Binding

**FIGURE 3:**
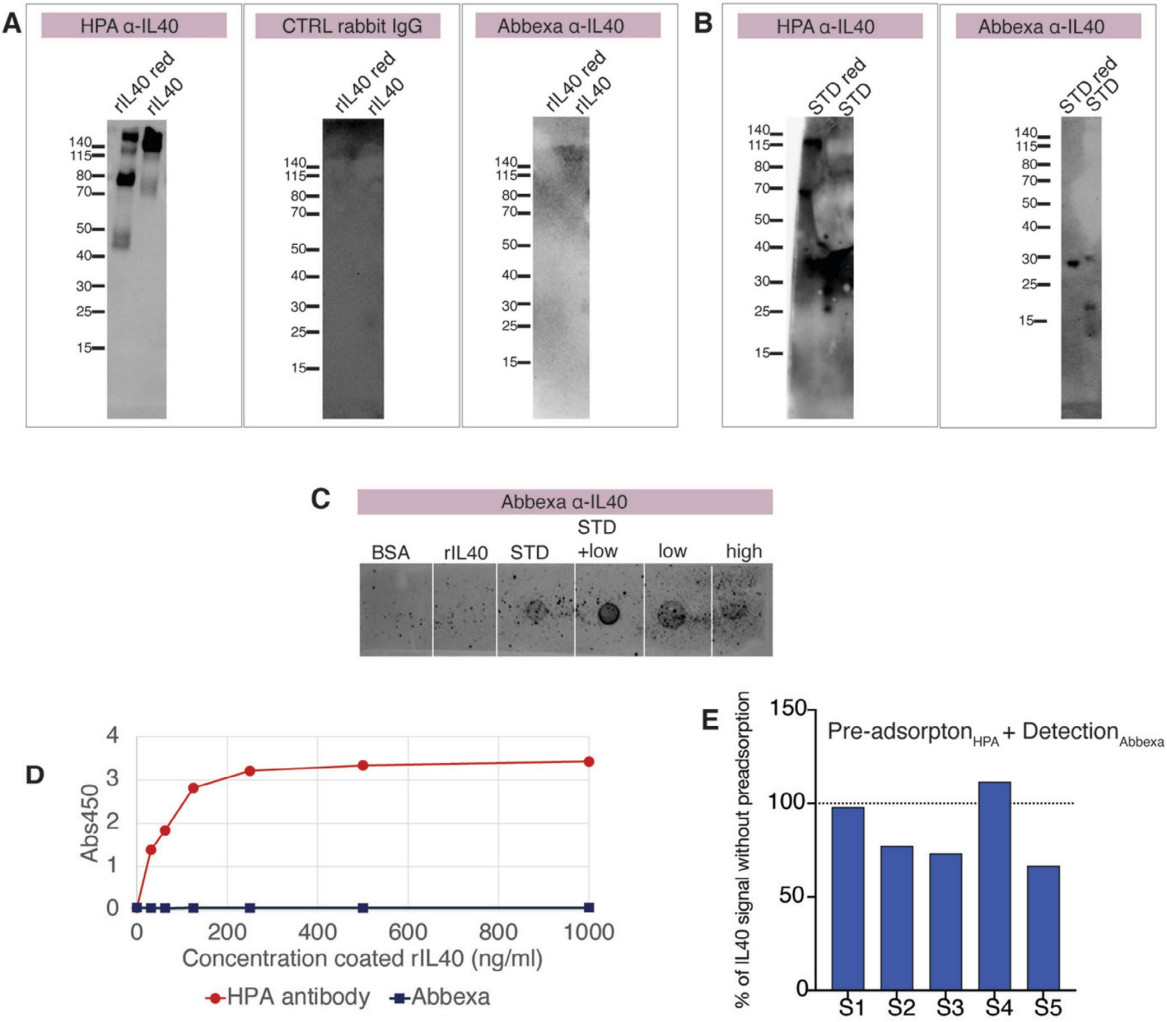
The Abbexa antibody detects protein in the assay standard but not other recombinant IL40. The Abbexa detection antibody was compared to an anti‐IL40 rabbit polyclonal antibody generated within the Human Protein Atlas. (A–C) IL40 was detected on Western blot using (i) anti‐IL40 (HPA029655) rabbit polyclonal and anti‐rabbit IgG HRP; (ii) control polyclonal rabbit IgG and anti‐rabbit IgG HRP; (iii) Abbexa (abx50853) anti‐IL40 detection reagent A and B diluted in the kit provides buffers (A). Detection of 20 ng of HEK293 expressed IL40‐Fc (rIL40) separated on SDS‐PAGE under reducing (red) and non‐reducing conditions. (B) Detection of equivalent to 10 ng Abbexa standard (abx50853). Two vials of 100 ng/vial were dissolved in 125 μL, buffer exchanged to PBS using Amicon Ultra (Sigma‐Aldrich) and 6.5 μL loaded on the gel (C) 20 ng recombinant IL40, 20 ng Abbexa standard, 20 ng Abbexa standard plus 2 μL plasma, or only plasma from an individual with high IL40 level by Abbexa ELISA (high) or low IL40 levels (low) were added to a nitrocellulose membrane for dot blot and detection with Abbexa anti‐IL40 using the same protocol as for the Western blots. (D) Comparison of ELISA detection of coated recombinant IL40 using anti‐IL40 (HPA029655) rabbit polyclonal or Abbexa anti‐IL40 detection A, and anti‐rabbit‐HRP (Cell Signaling Technology). (E) Results from Abbexa IL40 detection in five RA plasma samples after 1 h pre‐adsorption to an HPA anti‐IL40 coated plate.

The Abbexa detection antibody was further evaluated for binding in both dot blot and Western blot and showed, in similarity to the ELISA results, no detection of the recombinant IL40 (Fc‐IL40, theoretical MW 56 kDa, observed MW ~70 kDa) (Figure 3A). Purity and MW of the recombinant Fc‐IL40 was confirmed by SDS‐PAGE (Figure S6). No detection was seen also when the recombinant IL40‐Fc was coated directly to wells at high concentration (Figure 3D). The antibody did, however, show some detection of the kit provided IL40 reference in both Western blot and dot blot, which validated the detection setup of the Abbexa assay (Figure 3B,C). For these experiments, the lyophilized Abbexa reference was dissolved at a higher concentration in PBS. This resulted also in a very high concentration of carrier protein, which affected the SDS‐PAGE separation, but we could still detect a band of approximately correct size (30 kDa). Peculiarly, additional smaller sized band (~20 kDa) were also detected under reducing condition.

To ensure the quality of the recombinant IL40 we also evaluated binding of an antibody produced by Human Protein Atlas [3], rabbit polyclonal anti‐IL40 HPA029655, which was generated towards a defined IL40 epitope (residues 182–265 in the C‐terminal tail). Notably, the HPA antibody has not passed all quality control evaluation steps and is not commercially available. Nevertheless, it readily detected recombinant IL40 in both ELISA and Western blot (Figure 3A,D). Physiological IL40 has a predicted molecular weight of 27 kDa after processing of 20 amino acid long secretory signal. However, when comparing the HPA and Abbexa antibodies in Western blot of plasma samples, neither antibody detected any specific band of the right size in human plasma. Yet, it should be acknowledged that due to the high protein content, only a small volume of plasma can be separated per lane on SDS‐PAGE and the theoretical amount of IL40 may be under the limit of detection. Moreover, neither antibody is validated for Western blot (Figure 3A, S7). The Abbexa detection reagents stained plasma in dot blot, but there was no correlation with determined IL40 levels, as patient samples with low or high Abbexa IL40 levels were similar in signal (Figure 3C), suggesting unspecific binding of either the primary or secondary step.

No difference was observed in the Abbexa detection signal for plasma samples with high versus low IL40 concentrations (determined based on the Abbexa ELISA kit) (Figure S7). Interestingly, while the partially non‐validated HPA anti‐IL40 clearly binds HEK293‐cell produced recombinant IL40 independent of conformation, we could not see any detection of the Abbexa provided IL40 reference protein by ELISA (Figure 3D). However, there was potentially weak binding of the HPA antibody to something in the Abbexa reference standard by Western blot seen at longer exposure times (Figure 3B). Although, as discussed above, the high amount of carrier protein in the reference standard made the results hard to evaluate. When coating with the HPA anti‐IL40 antibody and continuing the assay with protocol and reagents from Abbexa, we did not see any detection of the Abbexa reference standard or could detect any signal in plasma (Figure S8). Moreover, plasma pre‐adsorption on an HPA anti‐IL40 coated plate showed only minor reduction of signal in the Abbexa assay (Figure 3E). Hence, the HPA and Abbexa antibodies do not seem to detect the same epitope.

**FIGURE 4:**
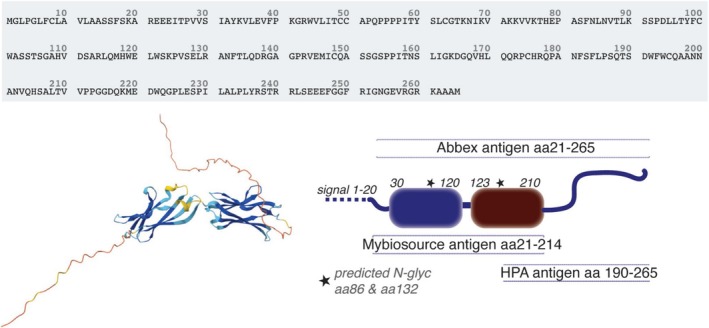
Schematic representation of the IL40 protein. IL40 protein sequence (Uniprot Q6UX52) and predicted structure by AlphaFold structure database [22]. The right panel displays information about where the evaluated antibodies in this study should bind.

In conclusion, using three different IL40 detection antibodies we find significant discrepancy in recognition which could implicate that one or several of these antibodies are not binding the same protein or alternatively recognising distinct structural forms of IL40 (Figure 4).

## 4 Discussion

Most known cytokines and chemokines were discovered more than 20 years ago, making the first description of IL40 in 2017 very recent. Since the discovery of IL40, a number of studies have focused on measuring serological levels of IL40 using commercially available ELISAs, with many using the ELISA provided by Mybiosource (MBS9337680) [2, 6, 7, 21]. Here we used another commercial ELISA provided by Abbexa (abx508530). To our surprise, we observed significant differences in the IL40 levels detected by the Abbexa assay compared to what has previously been reported using the Mybiosource assay. IL40 levels determined by the Abbexa assay showed similar plasma IL40 concentrations for healthy control populations compared to patients with rheumatic disease, including RA patients close to RA diagnosis (early RA), RA patients with B cell lymphoma as a comorbidity (RA‐L), and patients with ANCA‐associated vasculitis (AAV). Strikingly, IL40 concentrations were high both in patients with rheumatic disease and healthy donors. These results contrast with previous studies utilising the Mybiosource assay (MBS9337680), in which IL40 levels were observed to be significantly elevated in serum from patients with rheumatic disease.

Importantly, to ensure that the high IL40 measurements in healthy donors by the Abbexa assay were not due to underlying inflammation, we also measured serum levels of C‐reactive protein (CRP) which, as expected, were low in the healthy donors. Moreover, the analysis of three independent RA patient populations, including early untreated RA, limited the risk of sample selection bias. We included RA patients with lymphoma as a comorbidity, as IL40 has been shown to be produced in vitro by certain B cell lymphoma cell lines [1], thereby raising the question of whether IL40 could be elevated in RA patients with lymphoma. However, no significant difference was observed for RA‐L patients compared to any of the other two RA patient groups, healthy donors, or AAV patients. The only difference observed was lower levels in the patients with established RA without lymphoma as a co‐morbidity.

Notably, we observed large differences in IL40 measurements in plasma versus serum for both the Mybiosource assay and the Abbexa assay. At the same dilution, IL40 measurements were below the detection of the Mybiosource assay in plasma, whereas the opposite was observed for the Abbexa assay (e.g., IL40 levels below detection in serum). When comparing the correlation patterns for the concentration of IL40 in serum (Mybiosource assay) and IL40 in plasma (Abbexa assay), clear differences in the correlation patterns of IL40 with other cytokines/chemokines and hallmark RA‐associated autoantibodies were observed. The results show a discrepancy between two commercial assays claiming to specifically and quantitatively detect human IL40/C17ORF99 in serum and plasma samples. Notably, the lack of extensive validation and quality control of commercial antibodies and assays is a well‐known problem, and the discrepancies may be explained by off‐target interactions. Since the molecular data and regulation for IL40 is still relatively sparse, it is challenging to assess the validity of specific reagents. Thereby, highlighting the question of what the two different commercial assays are detecting, whether IL40 is truly being measured, or alternatively whether there are different forms of IL40 being detected. In this study, we suggest that the Abbexa (abx508530) assay captures an additional conformational form of IL40, which the Mybiosource assay (MBS9337680) does not capture.

The pre‐adsorption experiment showed that the monoclonal antibody used as capture on the Mybiosource assay plate binds the same IL40 as the Abbexa assay. Interestingly, pre‐incubation on the Abbexa assay and detection by the Mybiosource assay showed nearly unaltered concentrations of IL40 compared to paired samples that were not preincubated. Since we observed similar capture of IL40 for both assays, the results suggest that there is an additional conformational form of IL40 which is not detected by the Mybiosource assay but is detected by the Abbexa assay. This would further perhaps explain the discrepancies in IL40 measurements when using the two different commercial assays, such as the markedly different range of levels of IL40 reported in RA and healthy donors (12–22.2 and 1.0–1.9 ng/mL, respectively) in a previous study that used the Mybiosource assay, and the levels of IL40 we observed in RA patients and healthy donors (1.9–134.20 and 1.9–177.38 ng/mL, respectively) in our study using the Abbexa assay. Whether there are differences due to off‐target effects or due to different conformational forms, it is important to critically assess the validity of commercial assays to better understand what is being measured and whether this is representative of physiological IL40 and whether these can give insight into IL40 biology.

To better understand whether the Abbexa ELISA is detecting an additional form of IL40, we performed Western blot and dot blot to evaluate the binding. Consistent with the ELISA results, the Abbexa detection antibody did not bind the recombinant IL40 protein. Importantly, neither assay provided by Mybiosource or Abbexa could detect the mammalian produced recombinant IL40 protein, or even the respective IL40 reference by ELISA. The recombinant mammalian IL40 was produced as an Fc‐fusion protein but even when taking that into consideration, the molecular size by SDS‐PAGE and Western blot was significantly larger than the theoretical molecular weight (70 kDa vs. 56 kDa), which suggests post‐translational modifications. On the other hand, both IL40 kit references were produced in *E. coli*; thereby, the incompatibility between the two assays cannot be explained by posttranslational modifications. Importantly, the mammalian expressed recombinant IL40 was readily detected by a partially validated independent polyclonal rabbit antibody generated by the Human Protein Atlas, by both ELISA (in‐house) and Western blot, but could not detect the Abbexa IL40 kit reference.

The Abbexa detection antibody did, however, detect a protein of the correct size in Western blot of the kit provided standard, which would surprisingly indicate that the binding is not conformation dependent. Under reducing condition, where disulfide (cysteine) bonds are disrupted, a smaller size bands around 20 kDa and 8 kDa, which could be degraded/cleaved forms, but since the standard vial also contains carrier protein(s), this is hard to evaluate. It should be noted that the manufacturer has not provided any detailed information about the formulation or protein tags of the provided reference protein.

The HPA antibody is an independent polyclonal rabbit antibody obtained from the Human Protein Atlas by immunisation of a recombinant *human Protein* Epitope Signature Tags PrEST and affinity purification. The PrEST used to generate the HPA antibody primarily covers the AlphaFold predicted unstructured C‐terminal tail while the immunogen used for the Abbexa and Mybiosource includes the two predicted structured domains. For detection of IL40 in plasma, neither the HPA antibody nor the Abbexa detection antibody detected a band equivalent to the correct molecular weight for IL40. However, it should be emphasised that neither antibody was approved for Western blot and that the plasma concentration of IL40 is likely below the limit of detection. Importantly, the HPA anti‐IL40 antibody is not commercially available and did not pass the Western blot quality control. Moreover, according to the HPA record, the HPA anti‐IL40 immunohistochemistry evaluation showed high hepatocyte binding, which was not consistent with RNA expression data [20]. Nevertheless, the polyclonal HPA anti‐IL40 consistently showed high specific binding to recombinant IL40 by different methods in our studies.

Many cytokines are regulated by complex pre‐processing and multimerization mechanisms that generate functional or inactive forms. Examples include the TGFβ that requires release from a latent complex to function [23] and IL1β, IL18 and IL33 that require caspase cleavage of an inactive pro‐form to generate the mature active protein [24]. Several cytokines are not present as monomers in serum/plasma but are instead present as protein complexes (dimers, trimers etc.). Cytokines present as protein complexes include IL‐15 as a heterodimer [25], IL‐6 in complex with other receptors [26], BAFF as trimers and 60‐mer multimers [27] and TNF as a homotrimer [28]. To understand whether the discrepancies in IL40 measurements between the Abbexa assay (cat.no abx508530) and Mybiosource assay (MBS9337680) is due to differences in the detection of IL40 (e.g., detection of different forms of IL40) or due to off‐target effects, further validation studies are required to confirm the accurate detection of IL40 in physiological settings.

## Author Contributions

N.E., V.M., E.B. and C.G. conceptualised and designed the study and interpreted the data. N.E. and W.H. performed experiments and visualisation of data. P.H., A.J., E.H., L.K., I.G. and E.B. provided patient samples and clinical data and contributed with clinical expertise. N.E. performed statistical analysis. N.E. wrote the first manuscript draft. All authors were involved in revising the article critically and approved the final version of the manuscript.

## Funding

This work was supported by the the Swedish Research Council (2023‐02497), the Swedish Rheumatism Association (R‐1012541), King Gustaf V's 80‐year Foundation (FAI‐2023‐0956) and the Swedish Cancer Foundation (2010‐731, 2013‐456, 2016‐322).

## Ethics Statement

All participants gave informed consent, and the study was conducted in accordance with the ethical standards of the regional ethics review boards and followed the 1964 Helsinki declaration. Ethical approval was granted by the regional ethical committee for blood sampling and serology analyses of RA patients with lymphoma diagnosis (2009‐238), established RA patients (2009‐805‐31/4), early RA and healthy population control samples (1996‐174, 2006‐476, and 2021‐00125), and ANCA‐associated vasculitis (2008‐1143‐31 and 2022‐06448‐02).

## Conflicts of Interest

The authors declare no conflicts of interest.

## Supporting information


**Figure S1:** Correlations observed for IL40 plasma levels measured by the Mybiosource assay compared to the Abbexa assay.
**Figure S2:** Population controls have low inflammation according to CRP, which does not correlate with IL40 measurements.
**Figure S3:** Evaluation of hook effect and reproducibility after freeze thawing.
**Figure S4:** Correlation of IL40 by Mybiosource and other cytokine levels.
**Figure S5:** IL40 kit references are not recognised in the reciprocal ELISA.
**Figure S6:** Size of mammalian produced recombinant Fc‐IL40.
**Figure S7:** Western blot detection of IL40 in plasma.
**Figure S8:** Evaluation of cross‐recognition of IL40 by the HPA anti‐IL40 antibody and the Abbexa kit detection antibody.

## Data Availability

The data that support the findings of this study are available on request from the corresponding author. The data are not publicly available due to privacy or ethical restrictions.

## References

[1] J. Catalan‐Dibene , M. I. Vazquez , V. P. Luu , et al., “Identification of IL‐40, a Novel B Cell–Associated Cytokine,” Journal of Immunology 199 (2017): 3326–3335. 10.4049/jimmunol.1700534 PMC5667921 28978694

[2] A. Navrátilová , V. Bečvář , H. Hulejová , et al., “New Pro‐Inflammatory Cytokine IL‐40 Is Produced by Activated Neutrophils and Plays a Role in the Early Stages of Seropositive Rheumatoid Arthritis,” RMD Open 9 (2023): e002894. 37208028 10.1136/rmdopen-2022-002894 PMC10201233

[3] M. Uhlén , L. Fagerberg , B. M. Hallström , et al., “Tissue‐Based Map of the Human Proteome,” Science 347 (2015): 1260419. 25613900 10.1126/science.1260419

[4] “Tissue Expression of C17orf99 ‐ Summary ‐ the Human Protein Atlas,” accessed December 15, 2025, https://www.proteinatlas.org/ENSG00000187997‐C17orf99/tissue.

[5] S. Cai , X. Li , C. Zhang , et al., “Inhibition of Interleukin‐40 Prevents Multi‐Organ Damage During Sepsis by Blocking NETosis,” Critical Care 29 (2025): 29. 39819454 10.1186/s13054-025-05257-2 PMC11740647

[6] G. Guggino , C. Rizzo , L. Mohammadnezhad , et al., “Possible Role for IL‐40 and IL‐40‐Producing Cells in the Lymphocytic Infiltrated Salivary Glands of Patients With Primary Sjögren's Syndrome,” RMD Open 9 (2023): e002738. 37137540 10.1136/rmdopen-2022-002738 PMC10163598

[7] A. Navrátilová , L. Andrés Cerezo , H. Hulejová , et al., “IL‐40: A New B Cell‐Associated Cytokine Up‐Regulated in Rheumatoid Arthritis Decreases Following the Rituximab Therapy and Correlates With Disease Activity, Autoantibodies, and NETosis,” Frontiers in Immunology 12 (2021): 745523. 34745117 10.3389/fimmu.2021.745523 PMC8566875

[8] F. Dabbagh‐Gorjani , “A Comprehensive Review on the Role of Interleukin‐40 as a Biomarker for Diagnosing Inflammatory Diseases,” Autoimmune Diseases 2024 (2024): 1–8. 10.1155/2024/3968767 PMC10923619 38464677

[9] A. Navrátilová , S. Oreská , H. Wünsch , et al., “Serum IL‐40 Is Elevated in Systemic Sclerosis and Is Linked to Disease Activity, Gastrointestinal Involvement, Immune Regulation and Fibrotic Processes,” Arthritis Research & Therapy 27 (2025): 119. 40457473 10.1186/s13075-025-03570-3 PMC12128543

[10] L. A. Cerezo , A. Navrátilová , M. Kuklová , et al., “IL‐40 Is Up‐Regulated in the Synovial Fluid and Cartilage of Osteoarthritis Patients and Contributes to the Alteration of Chondrocytes Phenotype In Vitro,” Arthritis Research & Therapy 26 (2024): 146. 39080724 10.1186/s13075-024-03372-z PMC11289996

[11] P. J. Thul , L. Åkesson , M. Wiking , et al., “A Subcellular Map of the Human Proteome,” Science 356 (2017): eaal3321. 28495876 10.1126/science.aal3321

[12] “Expression of C17orf99 in Cancer ‐ Summary ‐ the Human Protein Atlas,” accessed December 15, 2025, https://www.proteinatlas.org/ENSG00000187997‐C17orf99/cancer.

[13] C. Bengtsson , A. Berglund , M.‐L. Serra , et al., “Non‐Participation in EIRA: A Population‐Based Case–Control Study of Rheumatoid Arthritis,” Scandinavian Journal of Rheumatology 39 (2010): 344–346. 20476868 10.3109/03009740903501634

[14] P. Stolt , C. Bengtsson , B. Nordmark , et al., “Quantification of the Influence of Cigarette Smoking on Rheumatoid Arthritis: Results From a Population Based Case‐Control Study, Using Incident Cases,” Annals of the Rheumatic Diseases 62 (2003): 835–841. 12922955 10.1136/ard.62.9.835 PMC1754669

[15] N. Euler , E. Hellbacher , E. A. Klint , et al., “Diffuse Large B Cell Lymphoma in Rheumatoid Arthritis Patients Is Associated With Elevated B‐Cell Driving Factors Including CXCL13,” Clinical Immunology 275 (2025): 110476. 40118130 10.1016/j.clim.2025.110476

[16] D. Aletaha , T. Neogi , A. J. Silman , et al., “Rheumatoid Arthritis Classification Criteria: An American College of Rheumatology/European League Against Rheumatism Collaborative Initiative,” Arthritis and Rheumatism 2010, no. 62 (2010): 2569–2581. 10.1002/art.27584 20872595

[17] C. Grönwall , K. Amara , U. Hardt , et al., “Autoreactivity to Malondialdehyde‐Modifications in Rheumatoid Arthritis Is Linked to Disease Activity and Synovial Pathogenesis,” Journal of Autoimmunity 84 (2017): 29–45. 28647488 10.1016/j.jaut.2017.06.004

[18] U. Hardt , A. Larsson , I. Gunnarsson , et al., “Autoimmune Reactivity to Malondialdehyde Adducts in Systemic Lupus Erythematosus Is Associated With Disease Activity and Nephritis,” Arthritis Research & Therapy 20 (2018): 36. 29482604 10.1186/s13075-018-1530-2 PMC5827973

[19] C. Grönwall , U. Hardt , J. T. Gustafsson , et al., “Depressed Serum IgM Levels in SLE Are Restricted to Defined Subgroups,” Clinical Immunology 183 (2017): 304–315. 28919518 10.1016/j.clim.2017.09.013

[20] “C17orf99 ‐ Antibodies ‐ the Human Protein Atlas,” accessed January 22, 2026, https://www.proteinatlas.org/ENSG00000187997‐C17orf99/summary/antibody.

[21] C. Rizzo , L. La Barbera , M. Lo Pizzo , et al., “Potential Involvement of IL‐40 in Kidney Disease Associated to Systemic Lupus Erythematosus,” Lupus 34 (2025): 1178–1183. 40677152 10.1177/09612033251361037

[22] J. Jumper , R. Evans , A. Pritzel , et al., “Highly Accurate Protein Structure Prediction With AlphaFold,” Nature 596 (2021): 583–589. 34265844 10.1038/s41586-021-03819-2 PMC8371605

[23] Z. Deng , T. Fan , C. Xiao , et al., “TGF‐β Signaling in Health, Disease, and Therapeutics,” Signal Transduction and Targeted Therapy 9 (2024): 61. 38514615 10.1038/s41392-024-01764-w PMC10958066

[24] W. P. Arend , G. Palmer , and C. Gabay , “IL‐1, IL‐18, and IL‐33 Families of Cytokines,” Immunological Reviews 223 (2008): 20–38. 18613828 10.1111/j.1600-065X.2008.00624.x

[25] C. Bergamaschi , J. Bear , M. Rosati , et al., “Circulating IL‐15 Exists as Heterodimeric Complex With Soluble IL‐15Rα in Human and Mouse Serum,” Blood 120 (2012): e1–e8. 22496150 10.1182/blood-2011-10-384362 PMC3390963

[26] P. Pignatti , L. Ciapponi , P. Galle , et al., “High Circulating Levels of Biologically Inactive IL‐6/SIL‐6 Receptor Complexes in Systemic Juvenile Idiopathic Arthritis: Evidence for Serum Factors Interfering With the Binding to gp130,” Clinical and Experimental Immunology 131 (2003): 355–363. 12562400 10.1046/j.1365-2249.2003.02052.x PMC1808632

[27] M. Lempicki , S. Paul , V. Serbulea , et al., “BAFF Antagonism via the BAFF Receptor 3 Binding Site Attenuates BAFF 60‐Mer‐Induced Classical NF‐κB Signaling and Metabolic Reprogramming of B Cells,” Cellular Immunology 381 (2022): 104603. 36182705 10.1016/j.cellimm.2022.104603 PMC10691782

[28] H. Daub , L. Traxler , F. Ismajli , B. Groitl , A. Itzen , and U. Rant , “The Trimer to Monomer Transition of Tumor Necrosis Factor‐Alpha Is a Dynamic Process That Is Significantly Altered by Therapeutic Antibodies,” Scientific Reports 10 (2020): 9265. 32518229 10.1038/s41598-020-66123-5 PMC7283243

