# Supplementary material for: IL40: A Newly Described Cytokine With Conflicting Measurements and Detection Variability—Are There Different Forms?
Source: Scand J Immunol. 2026 Mar 8;103(3):e70105. doi: 10.1111/sji.70105 (PMC12967690; doi:10.1111/sji.70105)
Supplement: Supplementary file 1 — Figure S1: Correlations observed for IL40 plasma levels measured by the Mybiosource assay compared to the Abbexa assay. Figure S2: Population controls have low inflammation according to CRP, which does not correlate with IL40 measurements. Figure S3: Evaluation of hook effect and reproducibility after freeze thawing. Figure S4: Correlation of IL40 by Mybiosource and other cytokine levels. Figure S5: IL40 kit references are not recognised in the reciprocal ELISA. Figure S6: Size of mammalian produced recombinant Fc‐IL40. Figure S7: Western blot detection of IL40 in plasma. Figure S8: Evaluation of cross‐recognition of IL40 by the HPA anti‐IL40 antibody and the Abbexa kit detection antibody. [file SJI-103-e70105-s001.pdf]

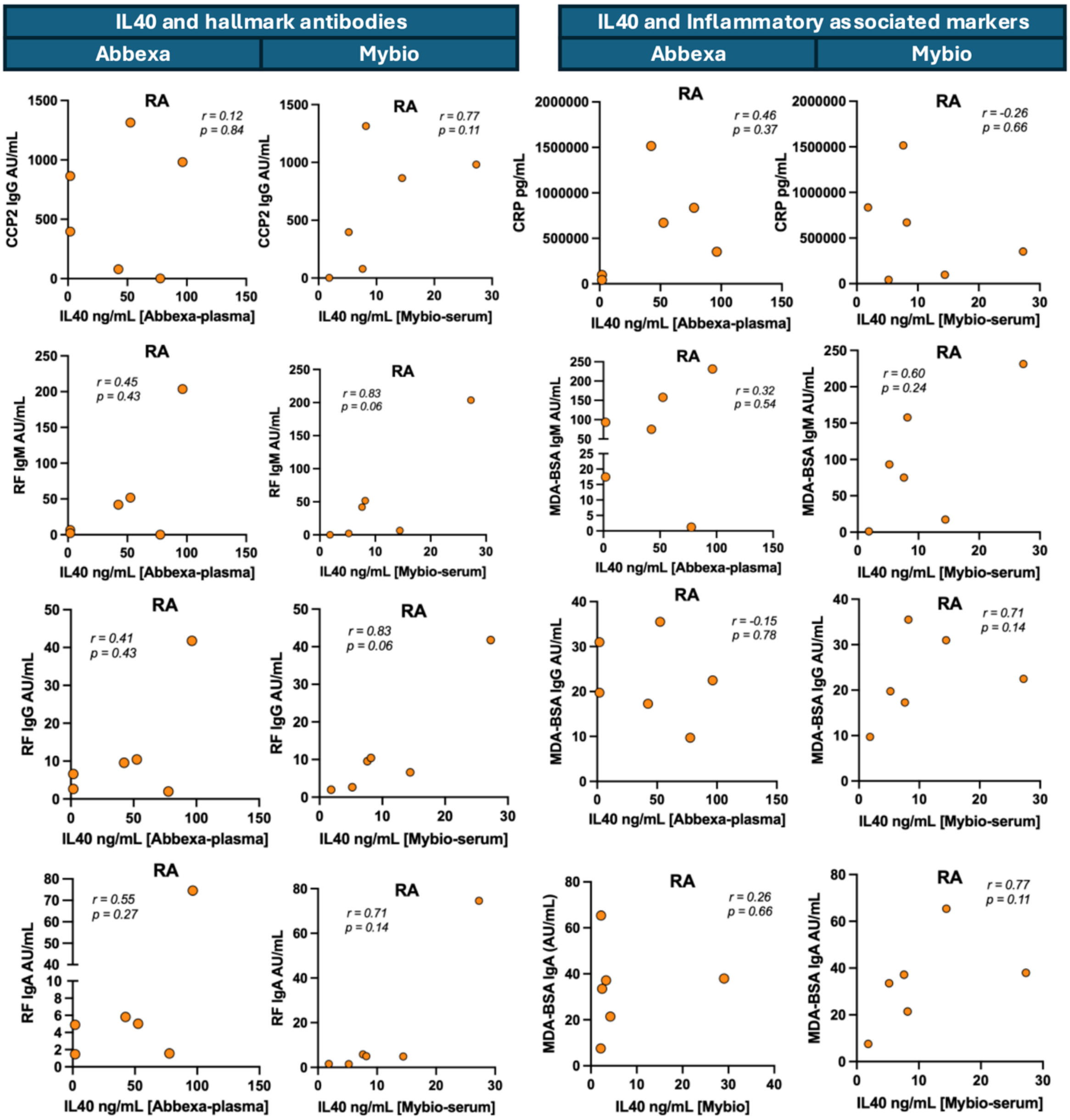


**Figure S1. Correlations observed for IL40 plasma levels measured by the Mybiosource assay compared to the Abbexa assay**

IL40 concentrations (ng/mL) by both the Abbexa assay (measured in plasma) and Mybiosource assay (measured in serum) have been determined for six RA plasma samples. Here we performed Spearman correlation analysis for IL40 measurements by both assays, with the concentration of different autoantibodies and C-reactive protein (CRP, pg/mL). For all statistical analysis, a p-value < 0.05 was considered significance. Significance written as p<0.05*, p<0.01**, p<0.001***.

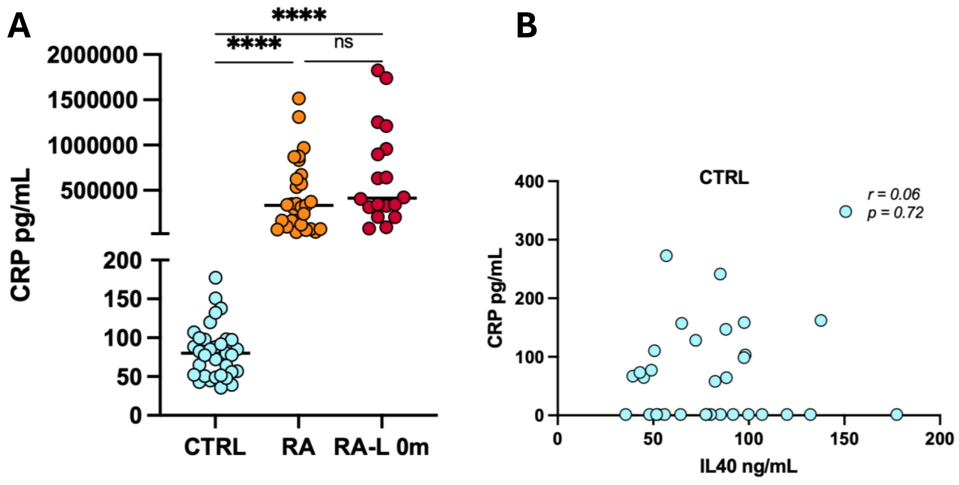


**Figure S2. Population controls have low inflammation according to CRP, which does not correlate with IL40 measurements**

(A) Serum CRP was measured using a high sensitivity ELISA (Thermo Fisher Scientific) in healthy donor population controls (CTRL) compared to rheumatoid arthritis patients with established disease (RA, n=29) and rheumatoid arthritis patients with B cell lymphoma as comorbidity (RA-L n=18) at baseline before lymphoma treatment (0m). Kruskal-Wallis test was performed for the statistical comparison. For all statistical analysis, a p-value < 0.05 was considered significance. Significance written as p<0.05*, p<0.01**, p<0.001***. (B) Spearman correlation between plasma IL40 levels and serum CRP in population controls.

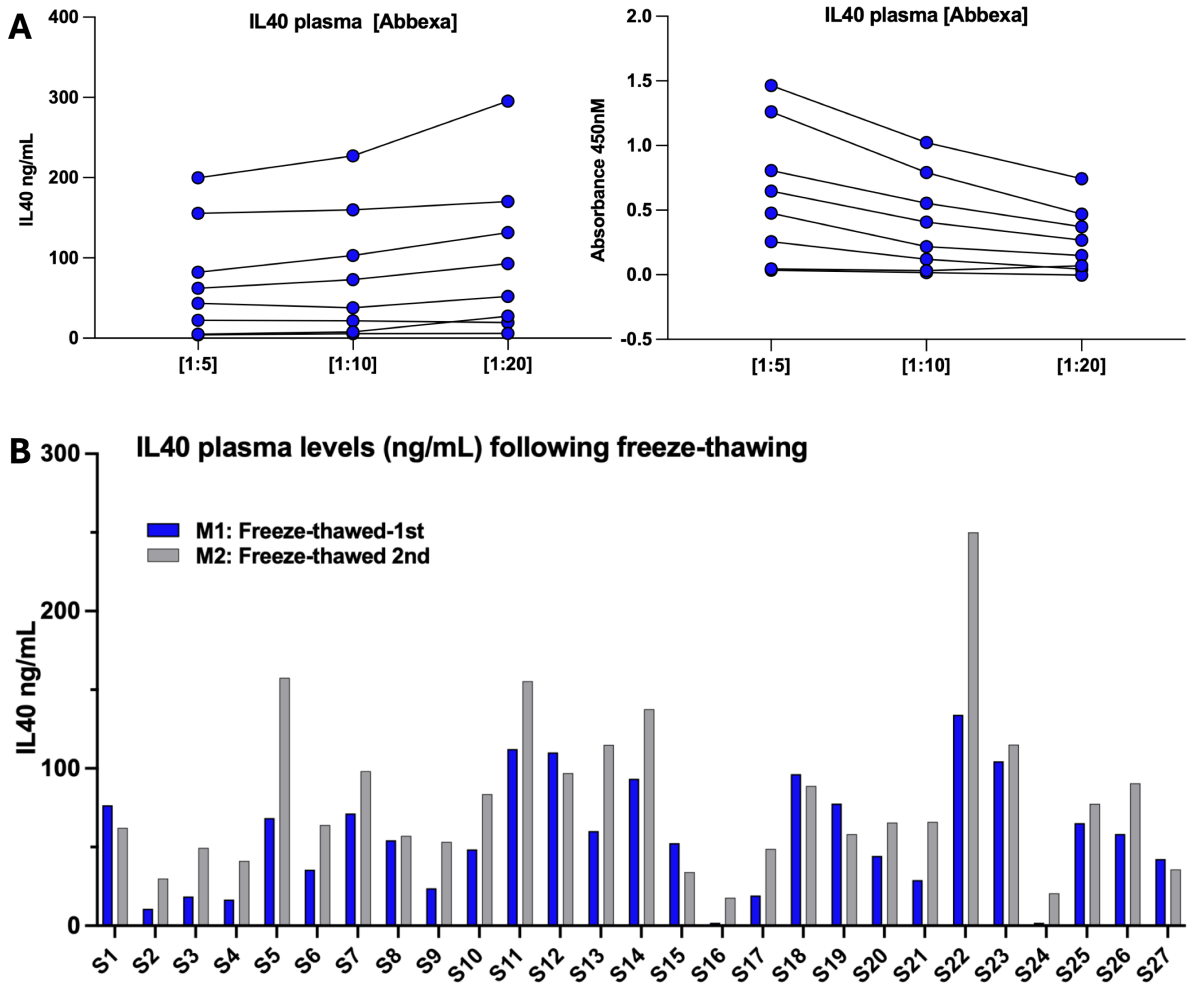


**Figure S3. Evaluation of hook effect and reproducibility after freeze thawing**

(A) Dilution of patient plasma and IL40 measurement to investigation whether the IL40 measurements obtained using the Abbexa assay in influenced by "hook effect". Plot demonstrates a serial dilution (1:5, 1:10, 1:20) of plasma samples from established rheumatoid arthritis patients (RA, n=6) and healthy donors (n=2) for detection of IL40 using Abbexa ELISA assay. (B) Comparison of IL40 at two independent IL40 measurements with the Abbexa assay (i) cryopreserved plasma samples stored in -80 with no or few previous freeze thaws, (ii) reanalysis after one cycle of freeze-thawing.

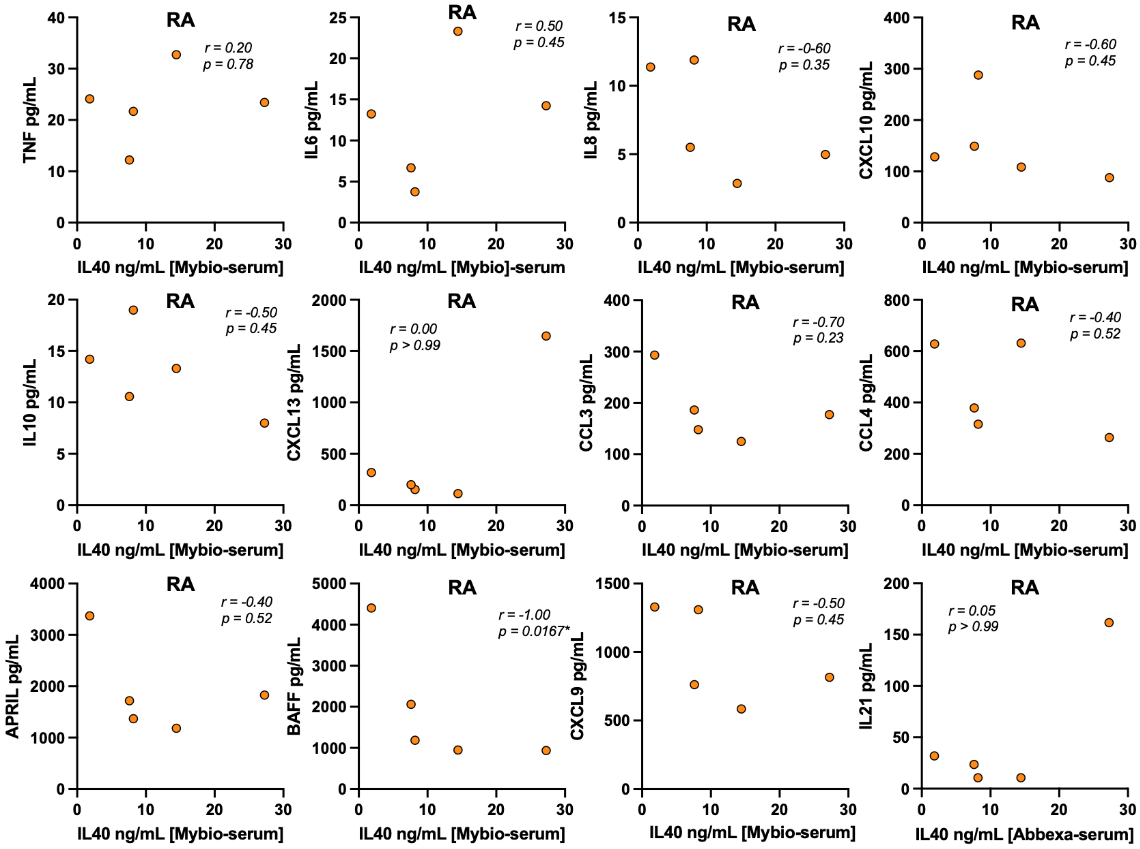


**Figure S4. Correlation of IL40 by Mybiosource and other cytokine levels**

Spearman correlation analysis for IL40 serum concentration (ng/mL) measurements by the Mybiosource assay (Mybio) and twelve different cytokines and chemokines measured by 12-plex Luminex assay in plasma from six RA patients with established disease.

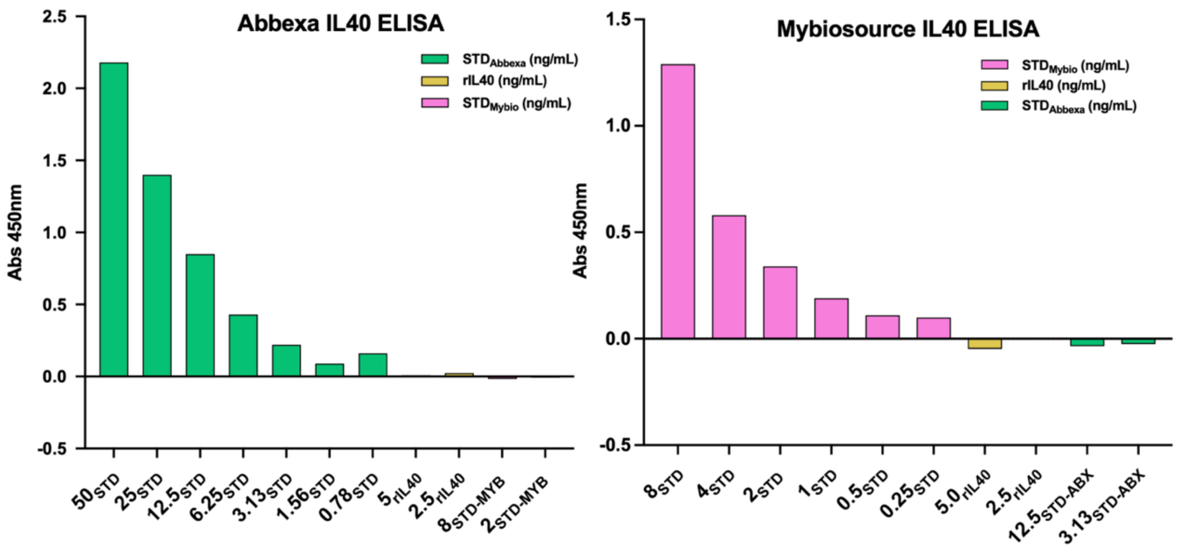


**Figure S5. IL40 kit references are not recognized in the reciprocal ELISA assay**

The absorption (450nM) for the kit IL40 standard reference for Abbexa (1.56 – 50 ng/mL, two-fold dilution) and Mybiosource (0.25 – 8 ng/mL, two-fold dilution) according to the recommended concentration for respective ELISA assay (green for Abbexa standard, pink for Mybiosource standard). The absorbance for the reciprocal standard reference and mammalian produced recombinant IL40 (rIL40) was also measured in each assay.

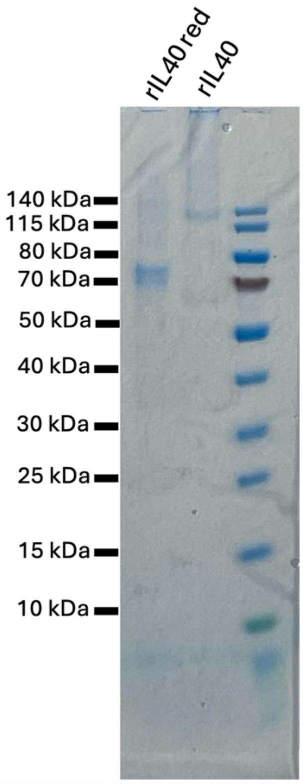


**Figure S6. Size of mammalian produced recombinant Fc-IL40.**

SDS-PAGE for recombinant Fc-conjugated IL40 (rIL40, Creative Biomart) produced in a mammalian cell line. Run under reduced (red) and non-reduced conditions. In both conditions rIL40 was run at 400 ng per lane.

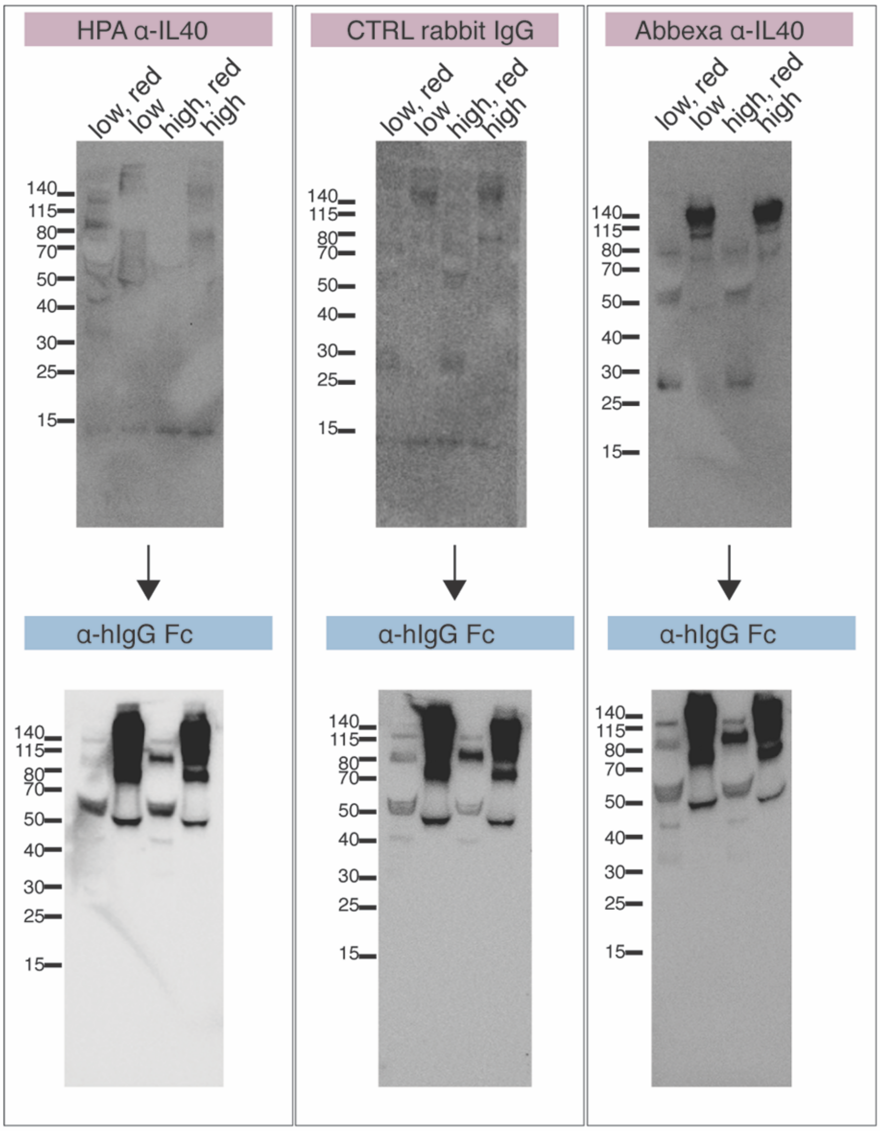


**Figure S7. Western blot detection of IL40 in plasma**

Western blot detection of proteins in plasma. 4 µl (1:20 diluted) of plasma from one patient with low IL40 by Abbexa detection (Low) and one patient with high IL40 levels (High) were separated on SDS-PAGE (Bolt 4-12% bis-tris gel, MES running buffer, transfer to PDVF membrane, Thermo Fisher Scientific). Detection using i) anti-IL40 (HPA029655) rabbit polyclonal and anti-rabbit IgG HRP (Cell Signaling Technology); ii) control chrompure polyclonal rabbit IgG (Jackson ImmunoResearch) and anti-rabbit IgG HRP; iii) Abbexa (abx50853) anti-IL40 detection reagent A and B diluted in the kit provides buffers. The bottom panels show loading control staining of the same membranes with rabbit anti-human IgG (Fc) HRP (Jackson ImmunoResearch). Red: reducing condition.

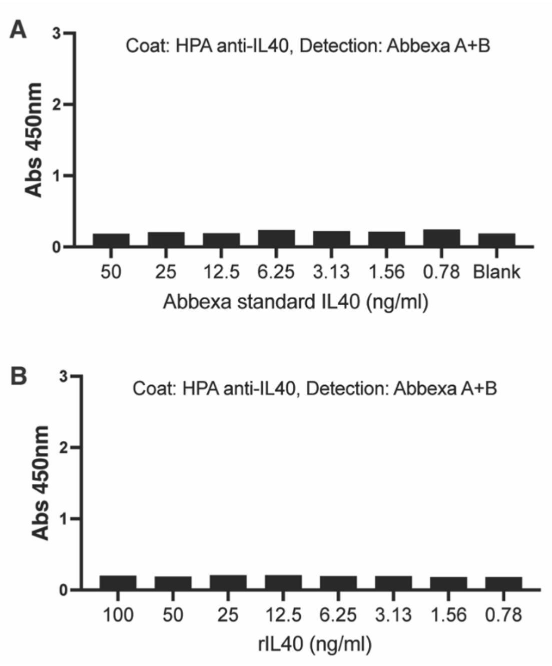


**Figure S8. Evaluation of cross-recognition of IL40 by the HPA anti-IL40 antibody and the Abbexa kit detection antibody**

Detection of the Abbexa provided reference sample (A) and (B) the HEK cell produced recombinant IL40 (rIL40, Creative Biomart). The assay was preformed using protocol and reagents provided by the Abbexa IL40 kit (abx508530) in all step with the exception of the that the samples were added to a high-binding ELISA plate coated with rabbit anti-IL40 from the human protein atlas (HPA029655, 1:300 in PBS over night, followed by 3% BSA blocking) instead of the provided kit plate.
